# When is a Ketogenic Diet Ketogenic? Comment on “Satiating Effect of a Ketogenic Diet and Its Impact on Muscle Improvement and Oxidation State in Multiple Sclerosis Patients, *Nutrients* 2019, *11*, 1156”

**DOI:** 10.3390/nu11081909

**Published:** 2019-08-15

**Authors:** Rainer Johannes Klement

**Affiliations:** Department of Radiotherapy and Radiation Oncology, Leopoldina Hospital Schweinfurt, Robert-Koch-Straße 10, 97422 Schweinfurt, Germany; rainer_klement@gmx.de; Tel.: +49-9721-7202761

**Keywords:** Ketogenic diet, Multiple sclerosis

Dear Editor,

Benlloch et al. recently published an article titled “Satiating Effect of a Ketogenic Diet and Its Impact on Muscle Improvement and Oxidation State in Multiple Sclerosis Patients” [1]. The authors showed that a Mediterranean diet supplemented with 2 × 30 mL coconut oil daily led to significant increases of satiety at lunch and dinner and improvements of body composition in 27 multiple sclerosis patients. The authors attributed these changes mainly to elevations of the ketone body beta-hydroxybutyrate (BHB) (see their Figure 2). However, I doubt that this conclusion is supported by the data because the study unfortunately suffers from methodological problems.

1. The title and premise of the study are misleading, as the putative ketogenic diet (KD) did not conform to the typical definition of a KD based on its macronutrient compositions [2,3]. It provided 40% energy from carbohydrates and only 40% from fat, which would be too high and low, respectively, to induce nutritional ketosis, defined as serum beta-hydroxybutydare (BHB) levels exceeding 0.5 mmol/L [4,5].
2. The authors tried to induce ketogenesis by providing 30 mL coconut oil for breakfast and 30 mL for lunch each day. Hence, the term “ketogenic” diet might in principle be justified. However, the data cannot provide such justification because not even one postprandial measurement was undertaken to measure the putative increase in postprandial BHB concentration. Vandenberghe et al. [6] showed that 20 mL of coconut oil provided together with a mixed meal (breakfast) did not significantly stimulate ketosis—only when given without an additional meal did coconut oil induce a mild (<0.5 mmol/L) increase in BHB concentrations.
3. Patients were advised to eat five meals daily. This tended to minimize intermittent fasting periods during the day and counteracted the entry into a postabsorptive state in which insulin levels are minimized.
4. Indeed, the fasting serum BHB concentrations measured after 4 months were only 0.1 ± 0.1 mmol/L. Such levels are not unusual after an overnight fast on any diet. Although nominally significantly higher than pre-intervention BHB concentrations (0.06 ± 0.0.4 mmol/L), the *p*-value of 0.045 was not corrected for multiple testing and even if taken at face value offers only weak evidence against the null hypothesis of no pre–post difference [7].

Another dissonant point is that the authors referred to the BHB transporters MCT1 and MCT4 as medium-chain triglyceride transporters instead of the correct notation: monocarboxylate/monocarboxylic acid transporters [8]. While I compliment the authors for their efforts to help multiple sclerosis patients by combining two beneficial concepts—those of a Mediterranean diet and ketosis—such a combination has been proposed before as the “Spanish Ketogenic Mediterranean Diet” which provides <30 g carbohydrates per day and hence fits the common perception of a KD much better [9].
